# Relationships between activation level, knowledge, self-efficacy, and self-management behavior in heart failure patients discharged from rural hospitals

**DOI:** 10.12688/f1000research.6557.1

**Published:** 2015-06-11

**Authors:** Van Do, Lufei Young, Sue Barnason, Hoang Tran

**Affiliations:** 1 Department of Health Services Research and Administration, College of Public Health, University of Nebraska Medical Center, Omaha, NE, 68198, USA; 2 College of Nursing-Lincoln Division, University of Nebraska Medical Center, Omaha, NE, 68198, USA; 3 Department of Epidemiology, College of Public Health, University of Nebraska Medical Center, Omaha, NE, 68198, USA

**Keywords:** Rural Populations, Cardiac Failure, Patient Activation, Patient Engagement, Self-Efficacy, Self-Management, Knowledge

## Abstract

Non-adherence to self-management guidelines accounted for 50% of hospital readmissions in heart failure patients. Evidence showed that patient activation affects self-management behaviors in populations living with chronic conditions. The purpose of this study was to describe patient activation level and its relationship with knowledge, self-efficacy and self-management behaviors in heart failure patients discharged from rural hospitals. Our study populations were recruited from two hospitals in rural areas of Nebraska. We found that two-thirds of the participants reported low activation levels (e.g., taking no action to manage their heart failure condition). In addition, low patient activation levels were associated with inadequate heart failure knowledge (p=.005), low self-efficacy (p<.001) and low engagement in heart failure self-management behaviors (p<.001) after discharge from hospital.

Heart failure is a major public health problem in the United States and worldwide. In the United States, heart failure affected 5.1 million patients with 12–15 million office visits, 6.5 million hospital days and cost approximately 32 billion U.S. dollars in 2010 ^1^. Despite the recent declining trend of cardiovascular disease-related mortality in the US, the rehospitalization rate for heart failure patients remains 30% within 60–90 days after discharge ^2^. Rural populations exhibit higher prevalence of heart failure ^3^ and rural patients are more likely to be readmitted due to heart failure ^4, 5^ compared to those in urban areas.

Among all causes for heart failure-related readmission, non-adherence to self-management is the most common reason, accounting for 50% of readmissions in heart failure patients ^6, 7^. Patient self-management is one of the key concepts in the Chronic Care Model developed by Edward H. Wagner ^8^. Self-management behaviors refer to the practice of activities that individuals initiate and perform on their own behalf in the interest of maintaining life, health, continuing personal development, and well-being ^9^. Self-management behaviors in heart failure patients primarily involve monitoring daily weight, following a restricted sodium diet, fluid restriction, taking prescribed medications, exercising regularly, and keeping scheduled follow-up appointments ^4^.

Prior nationwide studies among adults with chronic diseases showed that a high level of patient knowledge, efficacy and activation level were associated with good self-management ^10– 12^ and ultimately led to fewer hospitalizations and emergency department visits ^10, 13^. Few studies have reported the relationship between disease specific knowledge, self-efficacy, patient activation and self-management behaviors in rural heart failure patients. Self-efficacy for heart failure self-management is defined as how confidently individuals can achieve specific functions or control various aspects of their heart failure ^14^.

Patient engagement with self-management is critical for heart failure patients. Patient activation is the concept that can be applied as a means to determine patient engagement with self-management. The patient’s activation level, which is measured by Hibbard’s Patient Activation Measure (PAM), reflects the degree to which the person is ready, willing and able to engage in managing her or his health conditions ^12, 15^. Based on the PAM score, a person’s activation level is graded into 4 levels, from low to high: (1) Patients in level one believe they are responsible for managing their health; (2) patients in level two feel confident and knowledgeable regarding managing their health; (3) patients in level three actively engage in managing their health; and (4) patients in level four consistently engage in activities to manage their health and maintain those actions even under stress. The advancement in patient activation levels reflects the progress of the patient from being a passive care receiver to a more confident care manager ^12, 13, 15^.

Considering the low engagement in self-management of patients living with chronic diseases in the rural areas ^16, 17^, it is critical to examine whether there are positive relationships between knowledge, self-efficacy, patient activation and self-management behaviors. To our knowledge, these relationships have not been well studied in the rural heart failure population. Our study will contribute knowledge in understanding the impact of patient activation on self-management, which could inform the development of effective interventions to promote self-management in rural heart failure population. For this purpose, our study populations were recruited from hospitals in rural areas of Nebraska.

## Conceptual framework

**Figure 1.**
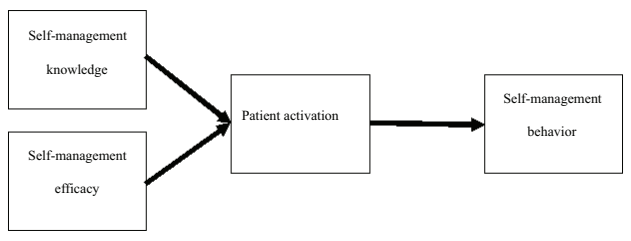
Patient activation concept framework.

As a cultural aspect, many rural patients endorse the importance of personal responsibility, productivity, and self-reliance in terms of health practice ^18^. Based on the rural culture belief and health practice, Bandura’s social cognitive theory ^19^, Hibbard’s Patient Activation Theory ^12, 20^, and the Chronic Care Model ^8, 21^, we proposed a conceptual framework ( Figure 1). Our framework assumes that by attaining self-management knowledge and efficacy, patients will advance their activation to higher levels, leading to long-term engagement in self-management behaviors.

## Methods

### Research design

We conducted a secondary analysis to evaluate the relationships between levels of patient activation and heart failure self-management knowledge, self-efficacy, and self-management behaviors in heart failure patients discharged from rural critical access hospitals to home. The data for this paper formed the baseline data from a NIH-funded randomized controlled trial titled “Patient AcTivated Care at Home (PATCH)” which aims to examine the feasibility of a 12-week home-based intervention to improve heart failure self-management adherence. This trial can be found on https://www.clinicaltrials.gov/ct2/show/NCT01964053.

### Sample and setting

Participants were recruited from October 2013 to December 2014 from two rural critical access hospitals in southeast Nebraska. Patients were eligible for the study if they: 1) were age 21 or older; 2) had heart failure as one of their discharge diagnoses; 3) had New York Heart Association (NYHA) class II to IV heart failure or had NYHA class I heart failure and had at least one heart failure-related hospitalization or emergency department visit in the previous year; 4) were discharged to home; 5) passed the Mini-Cog screen test screening for dementia ^22^; 6) understood English; and 7) had access to a phone.

We excluded patients who: 1) had depressive symptoms (received a score of 3 or above on the Patient Health Questionnaire-2 (PHQ-2) ^23^; 2) were diagnosed with liver cirrhosis; 3) were diagnosed with chronic renal failure; and 4) were diagnosed with other end stage and/or terminal illness (e.g. cancer) which limited the patient’s ability to perform self-management behaviors. The study setting is described in more detail in the study protocol ^24^.

### Measurements

We collected socio-demographic characteristics including age, gender, educational attainment, race/ethnicity, annual household income, marital status, and smoking status with a structured questionnaire. Clinical characteristics included comorbidities, echocardiographic ejection fraction (EF), and New York Heart Association (NYHA) Functional Classification.

We measured patient activation using the Short Form of the Patient Activation Measure (13-item version, available at http://www.ncbi.nlm.nih.gov/pmc/articles/PMC1361231/table/tbl1/) which has similar reliability and validity to the long form (22 item version) across different ages, genders and health condition status ^15^. Each item of the form was scored on the 5-point Likert response scale. For the ease of interpretation, the raw scores were transformed from the original metric to a 0–100 metric with higher scores indicating higher activation levels. Based on the patient activation score, patients were categorized into four levels: level 1 (score <47.0), level 2 (score 47.1–55.1), level 3 (score 55.2–67.0), and level 4 (score >67.0) ^12, 15^.

We measured self-efficacy using the Self-Care of Heart Failure Index (SCHFI) which has six questions on a 4 point Likert scale ^14^. The SCHFI was used to access the degree of achievement in self-care maintenance and management ^25^. The raw scores were standardized to a 100 point scale, with higher scores indicating higher efficacy. This measurement tool is reliable to assess self-efficacy with Cronbach’s alpha coefficient of .83 ^14^.

We measured patients’ heart failure knowledge using the 27 multiple-choice questions of the Atlanta Heart Failure Knowledge Test (AHFKT) ^26^. Each correct answer scored 1 point and the total score ranged from 0 to 27. This questionnaire established high reliability to evaluate heart failure knowledge with a Cronbach alpha coefficient of .84 ^26^.

We used the 29-item Revised Heart Failure Self-Care Behavior Scale (RHFSCS) to assess patients’ behavior in six heart failure care domains: (1) seeking medical help, (2) being aware of the effects of heart failure, (3) prevention of complications, (4) awareness of deleterious effects of medical care, (5) accepting heart failure, and (6) learning to live with heart failure ^27^. Each response is granted a score from 0 (none of the time) to 5 (all of the time) ^27^. The internal reliability of this questionnaire is consistent with a Cronbach alpha coefficient of .84 ^27^.

### Statistical analysis

Statistical analyses were conducted using SPSS version 20, with a p value less than .05 considered significant. We reported mean and standard deviation for continuous variables. Frequency and percentage were used to report categorical variables. Since patient activation level was an ordered variable, the Chi-square test for trend was used to assess correlations between four activation levels and the categorical variables (e.g. patient’s demographic and clinical characteristics). To compare continuous variables (e.g. behaviors, heart failure knowledge, Self-efficacy) across four activation levels, we used the ANOVA test for normally-distributed variables and the Kruskal-Wallis test for the non-normally distributed variables.

### Ethics

The study protocol was approved by the University of Nebraska Medical Center Institutional Review Board (IRB) and hospital ethical committees (IRB PROTOCOL # 228-13-EP).

## Results

### Demographic and clinical characteristics

There were 101 patients enrolled in this study. Table 1 describes socio-demographic and clinical characteristics. The mean age was 70±12.1 years. The majority of participants were women (63%). Two-thirds of participants had an annual household income of less than $50,000 or an educational attainment of high school graduate or lower. All the patients had multiple comorbidities including more than 2 of these chronic conditions: Hypertension (98.0%), Coronary artery disease (94.1%), Dyslipidemia (83.2%), Diabetes mellitus (41.6%), COPD/asthma (39.4%), CVA/stroke (17.8%), and cancer (6.9%). The majority of participants are at NYHA class II and III with the average EF of 55.8±11.1.

**Table 1.** Patient characteristics (n=101).

	N (%)	PAM score (SD)	p-value for the correlations with patient activation score
**Gender**			.806
Male	37 (36.6%)	58 (18.5)	
Female	64 (63.4%)	56 (18.9)	
**Age** ^$^			.224
65 years or younger	39 (38.6%)	60 (18.3)	
Older than 65 years	62 (61.4%)	55 (18.9)	
**Race/ Ethnicity**			.121
Caucasian	96 (95.0%)	58 (18.9)	
Non-Caucasian	5 (5.0%)	44 (6.5)	
**Educational attainment**			**.030**
Lower or High school graduate	64 (63.4%)	54 (17.6)	
Beyond high school	37 (36.6%)	62 (19.7)	
**Marital status**			.637
Married/Lived with partner	51 (50.5%)	58 (17.5)	
Not married/lived with partner	50 (49.5%)	56 (20.0)	
**Annual household income ^†^**			.400
Under $50,000	67 (71.3%)	57 (19.8)	
$50,000 – $75,000	9 (8.9%)	63 (17.9)	
Greater than $75,000	8 (7.9%)	61 (14.9)	
Refuse to response	10 (9.9%)	49 (14.9)	
**Smoking status**			.772
Not smoking	88 (87.1%)	57 (18.7)	
Current smoking	13 (12.9%)	56 (19.4)	
**New York Heart Classification**			.083
Class I	9 (8.9%)	58 (16.9)	
Class II	49 (48.5%)	61 (19.9)	
Class III	43 (42.6%)	52 (16.8)	
**Ejection fraction (SD) ^‡^**			.977
< 40%	9 (10.1%)	64 (24.7)	
≥ 40%	80 (89.9%)	57 (18.2)	
**Patient activation measure**			
Level 1	39 (39%)	--	--
Level 2	23 (23%)	--	--
Level 3	19 (19%)	--	--
Level 4	19 (19%)	--	--

**Significant results in bold face**

^$^missing data from 1 participant, n=100

^†^missing data from 7 participants, n=94

^‡^missing data from 12 participants, n=89

--: not applicable

### Patient activation level

The mean patient activation score was 57 and the median was 52 (95% CI 53–61). The patient activation score did not distribute normally with a positively skewed pattern. About 40% of participants believed that they were responsible for caring for their health and illness but failed to take action to manage their heart failure condition (level 1). A quarter of participants felt confident and knowledgeable enough to manage their health but failed to take action (level 2). Less than 40% of participants actually took some actions to manage their condition (level 3 and 4).

Patient activation levels were not significantly different across age group, gender, marital status, household income or smoking status. There was an association between activation level and educational attainment. Participants with educational attainment beyond high school had higher patient activation scores on the PAM compared to patients who only graduated high school or lower (62 vs. 54, p=.03).

### Patients’ self-management knowledge, efficacy and behavior

Using the Chi-square test for trend, we found an increasing trend between knowledge and self-efficacy across the four levels of patient activation. Patients with higher heart failure knowledge or greater self-efficacy were at a higher level of patient activation (p=.005 and p<.001, respectively). Patients who were at a higher level of patient activation also had higher scores of self-management behavior (p<.001). Table 2 describes the average score of self-management knowledge, efficacy, and behavior among 4 levels of patient activation level.

**Table 2.** Self-management knowledge, efficacy, and behavior among 4 levels of Patient activation level (n=100).

	Overall	Level 1	Level 2	Level 3	Level 4	p-value
	Mean (SD)					
Knowledge	20 (3.0)	20 (3.1)	19 (2.8)	21 (2.2)	22 (2.9)	.005 *
Efficacy	47 (23.8)	31 (18.5)	47 (16.4)	52 (17.8)	75 (18.4)	<.001 *
Behavior	89 (19.7)	82 (17.2)	93 (20.9)	91 (16.5)	98 (22.4)	<.001 *

**Significant results in bold face**

* df=1, Chi-square for trend

Heart failure (HF) patients discharged from rural hospitals: demographic, clinical, patient activation level, HF knowledge and self-management behavior characteristics Data was collected by questionnaires through patient interview and medical record review.
Details of labelling and abbreviation of the variables were included in the dataset ^40^.

## Discussion

This is one of the first studies to assess self-management knowledge, self-efficacy, activation level, and self-management behavior in American rural heart failure patients. The mean patient activation score of our study was 57, lower than that in Shively’s study (61.7) of heart failure patients or Green’s in the general chronic patient population (66.4) ^10, 28^. In addition, fewer of our rural heart failure participants were actively managing their condition compared to participants in other studies of both rural and urban areas ^10, 28^. According to our study, a lower percentage of heart failure patients (38%) had high activation levels and consistently took action to manage their health compared to diabetic patients in Begum’s (69.9%) and Rask’s studies (82.9%) ^29, 30^. However, patient activation scores in our study were higher than that in Evangelista’s study (37.3–39.3) in which the population consisted of heart failure patients in a palliative care setting ^31^. Similarly to Marshall’s study ^32^, the distribution of patient activation scores in our study was not different across various socio-demographic groups except for educational attainment. A higher educational attainment was associated with a higher level of activation in our patients. This finding was consistent with other studies which also found educational attainment to be the most powerful predictor for patient activation in the chronic disease population ^33– 36^. It is plausible that patients with higher educational levels are more likely to achieve better health literacy, giving them more awareness, skills and confidence to take self-management actions. In contrast to several studies, we did not find a variance of patient activation score among different age groups ^31, 37^. A possible explanation is that our participants seemed to be older and have narrower age variation (mean age 70, rank from 40 to 93) compared other studies (mean 53.7 ^31^ and age range from 18 to older than 75 ^37^). Evidence shows that an increase in patient activation level results in improved self-management behavior, leading to better health outcomes ^13, 38^. The overall low patient activation score in our heart failure participants indicates the need of developing interventions to enhance activation and self-management behavior in rural heart failure patients.

The associations between patient activation and heart failure knowledge, self-efficacy, and self-management behavior support our conceptual framework, as well as previous studies ^34, 37^. Patients with higher activation levels tended to have more knowledge to manage their heart failure, more confidence in self-management of heart failure, and were more likely to engage in self-management behaviors such as regular exercise, watching their diet and fluid intake, and adherence to medication. From a qualitative study, Dixon *et al.* reported that patients at lower activation levels indicated a lack of knowledge and lack of confidence as barriers for them to self-manage their health conditions ^39^. Our findings indicate that we can potentially boost patients’ activation by increasing their self-management knowledge and efficacy to take care of their own health. These findings confirmed our original hypothesis that strategies to enhance activation levels should be included in the intervention to promote heart failure self-management behaviors.

### Limitations

Our study had some limitations. We used only baseline data; therefore, we are unable to demonstrate the changes of patient activation scores, knowledge scores, self-efficacy and self-management behaviors over time. As a result, we could not establish the temporal relationship between knowledge, efficacy, patient activation level and their impact on behavior in this article, which will be reported in future manuscripts. Secondly, the patients who agreed to participate in the original randomized controlled trial might be more motivated than patients who refused to participate, therefore creating a pool of heart failure patients with higher activation levels compared to the general rural heart failure population. If this assumption is valid, the overall activation level in general rural heart failure population might be even lower than what is reported in this article, which indicates a great need to conduct interventions to enhance rural heart failure activation to engage in self-management behaviors.

### Clinical implication

Using the short version of PAM (13 items) is a feasible way to assess a patient’s activation level and identify those with low levels in both inpatient and outpatient settings. The assessment results allow clinicians to develop tailored interventions to support those high risk patients. Additionally, the patients with high activation levels could be encouraged and recruited as behavior coaches and/or peer supporters for those with low activation scores.

## Conclusions

Self-management plays a vital role in improving health outcomes and reducing healthcare costs in the heart failure population. Patient activation level is significantly associated with self-management behavior. However, the activation level in our rural heart failure patients was relatively low compared to patients in other studies, which suggest the need for interventions to improve activation levels in the rural heart failure population.

## Data availability

*F1000Research*: Dataset 1. Heart failure (HF) patients discharged from rural hospitals: demographic, clinical, patient activation level, HF knowledge and self-management behavior characteristics, 10.5256/f1000research.6557.d49205 ^40^

## Consent

Written informed consent for publication of clinical details was obtained from all the participants.

## References

[1] Go AS Mozaffarian D Roger VL: Heart disease and stroke statistics--2013 update: a report from the American Heart Association. *Circulation.* 2013;127(1):e6–e245. 10.1161/CIR.0b013e31828124ad 23239837 PMC5408511

[2] Gheorghiade M Vaduganathan M Fonarow GC: Rehospitalization for heart failure: problems and perspectives. *J Am Coll Cardiol.* 2013;61(4):391–403. 10.1016/j.jacc.2012.09.038 23219302

[3] Pearson TA Lewis C: Rural epidemiology: insights from a rural population laboratory. *Am J Epidemiol.* 1998;148(10):949–957. 9829866 10.1093/oxfordjournals.aje.a009571

[4] Jessup M Abraham WT Casey DE: 2009 focused update: ACCF/AHA Guidelines for the Diagnosis and Management of Heart Failure in Adults: a report of the American College of Cardiology Foundation/American Heart Association Task Force on Practice Guidelines: developed in collaboration with the International Society for Heart and Lung Transplantation. *Circulation.* 2009;119(14):1977–2016. 10.1161/CIRCULATIONAHA.109.192064 19324967

[5] Jin Y Quan H Cujec B: Rural and urban outcomes after hospitalization for congestive heart failure in Alberta, Canada. *J Card Fail.* 2003;9(4):278–285. 10.1054/jcaf.2003.43 13680548

[6] Evangelista LS Shinnick MA: What do we know about adherence and self-care? *J Cardiovasc Nurs.* 2008;23(3):250–257. 10.1097/01.JCN.0000317428.98844.4d 18437067 PMC2880251

[7] van der Wal MH Jaarsma T Moser DK: Qualitative examination of compliance in heart failure patients in The Netherlands. *Heart Lung.* 2010;39(2):121–130. 10.1016/j.hrtlng.2009.07.008 20207272

[8] Bodenheimer T Wagner EH Grumbach K: Improving primary care for patients with chronic illness. *JAMA.* 2002;288(14):1775–1779. 10.1001/jama.288.14.1775 12365965

[9] Orem DE Taylor SG Renpenning KM: Nursing: Concepts of Practice. 6th ed., Mosby St. Louis,2001 Reference Source

[10] Greene J Hibbard JH: Why does patient activation matter? An examination of the relationships between patient activation and health-related outcomes. *J Gen Internal Med.* 2012;27(5):520–526. 10.1007/s11606-011-1931-2 22127797 PMC3326094

[11] Mosen DM Schmittdiel J Hibbard J: Is patient activation associated with outcomes of care for adults with chronic conditions? *J Ambul Care Manage.* 2007;30(1):21–29. 10.1097/00004479-200701000-00005 17170635

[12] Hibbard JH Stockard J Mahoney ER: Development of the patient activation measure (PAM): Conceptualizing and measuring activation in patients and consumers. *Health Serv Res.* 2004;39(4 Pt 1):1005–1026. 10.1111/j.1475-6773.2004.00269.x 15230939 PMC1361049

[13] Hibbard JH Greene J Tusler M: Improving the outcomes of disease management by tailoring care to the patient's level of activation. *Am J Manag Care.* 2009;15(6):353–360. 19514801

[14] Riegel B Lee CS Dickson VV: An update on the self-care of heart failure index. *J Cardiovasc Nurs.* 2009;24(6):485–497. 10.1097/JCN.0b013e3181b4baa0 19786884 PMC2877913

[15] Hibbard JH Mahoney ER Stockard J: Development and testing of a short form of the patient activation measure. *Health Serv Res.* 2005;40(6 Pt 1):1918–1930. 10.1111/j.1475-6773.2005.00438.x 16336556 PMC1361231

[16] Bell RA Arcury TA Snively BM: Diabetes foot self-care practices in a rural triethnic population. *Diabetes Educ.* 2005;31(1):75–83. 10.1177/0145721704272859 15779248 PMC1613259

[17] Coronado GD Thompson B Tejeda S: Sociodemographic factors and self-management practices related to type 2 diabetes among Hispanics and non-Hispanic whites in a rural setting. *J Rural Health.* 2007;23(1):49–54. 10.1111/j.1748-0361.2006.00067.x 17300478

[18] Bardach SH Tarasenko YN Schoenberg NE: The role of social support in multiple morbidity: self-management among rural residents. *J Health Care Poor Underserved.* 2011;22(3):756–771. 10.1353/hpu.2011.0083 21841277 PMC3624890

[19] Bandura A: Perceived self-efficacy in cognitive development and functioning. *Educational Psychologist.* 1993;28(2):117–148. 10.1207/s15326985ep2802_3

[20] Hibbard JH Mahoney ER Stock R: Do increases in patient activation result in improved self-management behaviors? *Health Serv Res.* 2007;42(4):1443–1463. 10.1111/j.1475-6773.2006.00669.x 17610432 PMC1955271

[21] Wagner EH Austin BT Davis C: Improving chronic illness care: translating evidence into action. *Health Aff (Millwood).* 2001;20(6):64–78. 10.1377/hlthaff.20.6.64 11816692

[22] Borson S scanlan J Brush M: The mini-cog: a cognitive ‘vital signs’ measure for dementia screening in multi-lingual elderly. *Int J Geriatr Psychiatry.* 2000;15(11):1021–1027. 10.1002/1099-1166(200011)15:11<1021::AID-GPS234>3.0.CO;2-6 11113982

[23] Li C Friedman B Conwell Y: Validity of the Patient Health Questionnaire 2 (PHQ-2) in identifying major depression in older people. *J Am Geriatr Soc.* 2007;55(4):596–602. 10.1111/j.1532-5415.2007.01103.x 17397440

[24] Young L Barnason S Do V: Promoting self-management through adherence among heart failure patients discharged from rural hospitals: a study protocol [v2; ref status: indexed, http://f1000r.es/5c7]. *F1000Res.* 2015;3 10.12688/f1000research.5998.2 PMC4367517 25844160

[25] Riegel B Carlson B Moser DK: Psychometric testing of the self-care of heart failure index. *J Card Fail.* 2004;10(4):350–360. 10.1016/j.cardfail.2003.12.001 15309704

[26] Reilly CM Higgins M Smith A: Development, psychometric testing, and revision of the Atlanta Heart Failure Knowledge Test. *J Cardiovasc Nurs.* 2009;24(6):500–509. 10.1097/JCN.0b013e3181aff0b0 19858959 PMC2828039

[27] Artinian NT Magnan M Sloan M: Self-care behaviors among patients with heart failure. *Heart Lung.* 2002;31(3):161–172. 10.1067/mhl.2002.123672 12011807

[28] Shively MJ Gardetto NJ Kodiath MF: Effect of patient activation on self-management in patients with heart failure. *J Cardiovasc Nurs.* 2013;28(1):20–34. 10.1097/JCN.0b013e318239f9f9 22343209

[29] Begum N Donald M Ozolins IZ: Hospital admissions, emergency department utilisation and patient activation for self-management among people with diabetes. *Diabetes Res Clin Pract.* 2011;93(2):260–267. 10.1016/j.diabres.2011.05.031 21684030

[30] Rask KJ Ziemer DC Kohler SA: Patient activation is associated with healthy behaviors and ease in managing diabetes in an indigent population. *Diabetes Educ.* 2009;35(4):622–630. 10.1177/0145721709335004 19419972

[31] Evangelista LS Liao S Motie M: On-going palliative care enhances perceived control and patient activation and reduces symptom distress in patients with symptomatic heart failure: a pilot study. *Eur J Cardiovasc Nurs.* 2014;13(2):116–123. 10.1177/1474515114520766 24443421 PMC4455924

[32] Marshall R Beach MC Saha S: Patient activation and improved outcomes in HIV-infected patients. *J Gen Intern Med.* 2013;28(5):668–674. 10.1007/s11606-012-2307-y 23288378 PMC3631066

[33] Lubetkin EI Lu WH Gold MR: Levels and correlates of patient activation in health center settings: Building strategies for improving health outcomes. *J Health Care Poor Underserved.* 2010;21(3):796–808. 10.1353/hpu.0.0350 20693726

[34] Nijman J Hendriks M Brabers A: Patient activation and health literacy as predictors of health information use in a general sample of dutch health care consumers. *J Health Commun.* 2014;19(8):955–69. 10.1080/10810730.2013.837561 24397280

[35] Rademakers J Nijman J van der Hoek L: Measuring patient activation in The Netherlands: translation and validation of the American short form Patient Activation Measure (PAM13). *BMC Public Health.* 2012;12:577. 10.1186/1471-2458-12-577 22849664 PMC3490810

[36] Saha S Koley M Mahoney ER: Patient activation measures in a government homeopathic hospital in India. *J Evid Based Complementary Altern Med.* 2014;19(4):253–259. 10.1177/2156587214540175 24972592

[37] Hendriks M Rademakers J: Relationships between patient activation, disease-specific knowledge and health outcomes among people with diabetes; a survey study. *BMC Health Serv Res.* 2014;14(1):393. 10.1186/1472-6963-14-393 25227734 PMC4175625

[38] Hibbard JH Greene J: What the evidence shows about patient activation: better health outcomes and care experiences; fewer data on costs. *Health Aff (Millwood).* 2013;32(2):207–214. 10.1377/hlthaff.2012.1061 23381511

[39] Dixon A Hibbard J Tusler M: How do People with Different Levels of Activation Self-Manage their Chronic Conditions? *Patient.* 2009;2(4):257–268. 10.2165/11313790-000000000-00000 22273246

[40] Do V Young L Barnason S: Dataset 1 in: Relationships between activation level, knowledge, self-efficacy, and self-management behavior in heart failure patients discharged from rural hospitals. *F1000Research.* 2015 Data Source 10.12688/f1000research.6557.1 PMC4505779 26213616

