# Supplementary material for: Relationships between activation level, knowledge, self-efficacy, and self-management behavior in heart failure patients discharged from rural hospitals
Source: F1000Res. 2015 Jun 11;4:150. [Version 1] doi: 10.12688/f1000research.6557.1 (PMC4505779; doi:10.12688/f1000research.6557.1)
Supplement: Peer review history

# Peer review: Referee response for version 1

**DOI:** 10.5256/f1000research.7041.r9428

**Published:** 2015-07-09

**Authors:** George Sokos, Megan Clarke

**Affiliations:** 1 Cardiovascular Institute, Allegheny General Hospital, Pittsburgh, USA; 2 Section of Heart Failure/Transplant and Pulmonary Hypertension, Allegheny General Hospital, Pittsburgh, PA, USA

1. Page 2, paragraph 1: Consider adding information about CMS limited reimbursement for heart failure readmissions as this is a significant current concern in US hospitals.
2. Figure 1 and throughout entire paper:  Consider simplifying terms “self-management knowledge” to “Knowledge”,  “Self management efficacy” to “efficacy”. Many different variations of these terms are used throughout which reduces the readability of the paper. Table 2 on page 5 is organized well and has these terms outlined more simply.
3. Page 2, paragraph 3, last sentence: Consider changing “self-efficacy for heart failure self-management “ to “Efficacy for heart failure self-management” as the terms can be confusing if the reader is trying to understand the concept using Figure 1. This may be considered throughout the paper. 
4. Page 2, paragraph 4: It might be more helpful for the reader to see the PAM scores 1-4 in a table to allow for easy referencing when reading the paper.
5. Page 2, paragraph 5, first sentence: Consider changing “self-efficacy” to “efficacy”  (see comment number 2 above).
6. Page 2, paragraph 6, last sentence: Consider removing “long-term” as this specific study make correlations but does not address the length of time the patient’s behavior will be impacted.
7. Page 3, paragraph 1, sentence 1: Consider changing term “self efficacy” to efficacy (see comment number 2 above).
8. Page 3, paragraph 6, sentence 1: Consider changing term “self efficacy” to efficacy (see comment number 2 above).
9. Page 3, Measurement section: Consider arranging paragraphs to match flow in Figure 1 (Knowledge, Efficacy, Patient Activation, and Self-management behavior).
10. Page 3, paragraph 8: Change “patients behavior” to “patients self-management behavior”.
11. Table 1: Consider additional category of stage of heart failure (A,B,C,D) if available.
12. Page 5, paragraph 3 (first paragraph of discussion section): The statement regarding this study’s similarity to other findings which show educational attainment as the “most powerful predictor for patient activation” may led readers to believe that knowledge, efficacy, and behavior were compared and that education outweighed the others. I would consider re-wording and removing the term “most powerful.”.
13. Page 5, paragraph 4, sentence 2 (second paragraph of discussion section): Can “confidence” be changed to “efficacy” here to keep language consistent?
14. Page 5, paragraph 4, sentence 4: This sentence infers that the intervention in this study was to increase knowledge and efficacy to improve patient activation, but from my understanding this was a correlation and not an intervention.

We have read this submission. We believe that we have an appropriate level of expertise to confirm that it is of an acceptable scientific standard.

# Peer review: Referee response for version 1

**DOI:** 10.5256/f1000research.7041.r8999

**Published:** 2015-06-22

**Authors:** Hoa L. Nguyen

**Affiliations:** 1 Department of Epidemiology, Baylor Scott & White Health, Dallas, TX, USA

The paper potentially provides important information about the relationship between activation level, knowledge, self-efficacy and self-management behavior in the rural population with heart failure to inform future interventions.

I have several minor comments that the authors may consider in the revised version:

1. Abstract: The authors may consider adding sample size, and several basic characteristics of the study population (mean age, sex distribution, etc.) in the abstract.
2. Since the sample size of the study is relatively small (n=101), and the authors stated that the distribution of the patient activation score was skewed (page 3, last paragraph), they may consider reporting medians (inter quartile ranges) in the tables and text in addition to means (SDs).
3. Statistical analysis: The authors mentioned Chi-square, Anova, and Kruskal-Wallis tests were used to compare patients’ characteristics across 4 activation levels, but it is unclear where the results were reported in the results section.
  In table 1, patients’ characteristics for all patients were presented (not according to activation levels) and means (SDs) PAM score by patients characteristics were reported.
  The authors may consider stating clearly in the statistical analysis how they treated the activation score (the main outcome of interest) as continuous or as categorical variable or both approaches and report the results accordingly.
4. Discussion: The author wrote “the distribution of patient activation scores in our study was not different across various socio-demographic groups except for educational” (page 5). This may be due to small sample size issue; the study may be underpowered to detect potential differences (e.g. the mean activation score difference by race/ethnicity was 14 (58 vs. 44), however the difference was not statistically significant). This is one of the study limitations.

I have read this submission. I believe that I have an appropriate level of expertise to confirm that it is of an acceptable scientific standard.
